# Editorial: Molecular underpinnings of relapse-like behavior: drug context and development

**DOI:** 10.3389/fnbeh.2023.1347621

**Published:** 2024-01-04

**Authors:** Caitlin A. Orsini, Jee Hyun Kim, Amy A. Arguello

**Affiliations:** ^1^Department of Psychology, Neurology and Waggoner Center for Alcohol and Addiction Research, The University of Texas at Austin, Austin, TX, United States; ^2^IMPACT, The Institute for Mental and Physical Health and Clinical Translation, School of Medicine, Deakin University, Geelong, VIC, Australia; ^3^Department of Psychology, Behavioral Neuroscience, Interdisciplinary Science and Technology Building, Michigan State University, East Lansing, MI, United States

**Keywords:** relapse, cocaine self-administration, cocaine conditioned place preference, extinction, estrous cycle, risk-taking behavior, ethanol, adolescent

## Introduction

Substance use disorders (SUDs) comprise a complex set of behavioral phenomena that can detrimentally affect quality of life (Saunders, 2017). Patients diagnosed with SUDs can suffer from repeated relapse to drug use despite long periods of abstinence, increased craving when exposed to drug-associated paraphernalia or previous drug-taking environments, and long-lasting impairments in cognitive function (Gawin and Kleber, 1986; Guerin et al., 2021). Importantly, relapse behaviors are also observed in animals that receive or self-administer a drug, providing biological tools to understand the human condition (Perry et al., 2014). Therefore, the current Research Topic aims to gather insight from preclinical studies relevant to drug-taking and seeking to understand the factors underlying SUDs. The contributing articles describe the impact contextual stimuli have on relapse-like behavior, adolescent drug exposure effects on anxiety-like behavior, reversal learning, risk-based decision making, and the molecular mechanisms contributing to relapse-like behavior as a function of age and sex.

## The contributions

The investigation by Solecki et al. utilized both cocaine conditioned place preference (CPP) and self-administration procedures to investigate the role of ventral tegmental area (VTA) alpha1-adrenergic receptors in the acquisition of cocaine-seeking and cocaine-taking behaviors. The authors found that intra-VTA administration of prazosin (alpha1-adrenergic receptor antagonist) impaired acquisition of cocaine CPP but did not affect cocaine-taking behavior or ultrasonic vocalizations. Interestingly, intra-VTA administration of prazosin also blocked dopamine release in the nucleus accumbens, which implicates alpha1-adrenergic receptors in modulating the mesolimbic reward pathway. In their commentary, Lasne et al. highlight the relevance of the current findings to a growing understanding of the role of noradrenaline in cocaine-induced behavior and general reward-based learning. These findings are also translationally relevant; prazosin has been clinically trialed to determine if it reduces methamphetamine use, although the results are yet unpublished (St. Peters et al., 2023).

Behavioral treatment for SUDs mostly involves cue exposure therapy, which relies on extinction as its underlying process (Loeber et al., 2006). In the context of SUDs, extinction is the decrease in the emotional, behavioral, and cognitive reactivity to a drug-associated cue due to repeated exposure to the cue without any drug availability. The persistence of compulsive reward-seeking behavior after extinction poses a significant challenge in effectively treating SUDs. Extinction is sensitive to contextual shifts, with contexts that are different from where extinction occurred increasing reward-seeking, referred to as renewal. Renewal differs between males and females and is sensitive to hormonal fluctuations (Anderson and Petrovich, 2015, 2017; Hilz et al., 2019). Hilz et al. (2019) explored the neural substrates associated with hormonal modulation of renewal by mapping activity-induced mRNA activity. Females displayed greater renewal of food-seeking behavior when they underwent extinction training in the phase of the estrous cycle in which ovarian hormones are the highest and were tested for renewal in a different phase of the estrous cycle. Under these conditions, neurons in the prefrontal cortex, amygdala and hippocampus were activated to both the extinction context as well as the renewal (novel) context. Only in the dorsal region of the hippocampus did neuronal activation differ based on the context in which animals were tested. These findings provide valuable evidence for how hormonal fluctuations can modulate relapse-like behavior at the behavioral and neural levels and have important implications for improving the exposure therapy efficacy for women with SUD.

Truckenbrod et al. approach relapse from a different perspective, focusing on the cognitive mechanisms and their underlying hormonal substrates that may contribute to relapse after abstinence from cocaine use. Indeed, chronic psychostimulant exposure results in long-lasting impairments in decision making, such as risk taking, which can confer greater risk for relapse (Chen et al., 2020). In Truckenbrod et al., prior cocaine self-administration surprisingly did not alter risk taking when rats were forced to abstain. However, when the relationship between the magnitude of cocaine intake and subsequent risk-taking behavior was examined, greater cocaine intake predicted greater preference for the risky option in females but not in males. Moreover, hormonal cyclicity was significantly disrupted in females that self-administered cocaine, and this effect persisted well into abstinence. Collectively, these findings suggest that changes in risk-based decision making in females is determined by the magnitude of cocaine intake, which may be due cocaine-induced disruptions in the female endocrine system.

Early exposure to substances is also a critical factor that influences the life-long risk to develop SUDs. Matthews et al. investigated the impact of adolescent chronic intermittent ethanol exposure on spatial learning, anxiety-like behaviors, and molecular markers of stress across the lifespan. The authors found that male rats exposed to chronic intermittent ethanol had increased anxiety-like behaviors in the elevated plus maze when tested as adolescents, but this effect did not persist when rats were tested as adults or aged adults. Further, adolescent male rats exposed to ethanol exhibited impaired spatial reversal learning on a water maze task when they were tested as aged adults. Females did not show such behavioral changes due to ethanol exposure. Higher expression of activated microglia in the hippocampus was observed in female rats compared with males, but microglial changes related to ethanol were not observed in either sex. These findings suggest adolescent ethanol exposure has long-lasting impacts on specific forms of cognition and establish a basis for further work to examine how longer periods of chronic ethanol exposure or ethanol self-administration may impact emotion and cognition in aging.

Lastly, Arguello et al. provide a review of rodent studies that utilize cocaine CPP and cocaine self-administration to understand the impact of adolescent cocaine use on subsequent reward, craving and relapse-like behavior. Arguello et al. also describe the types of drug-associated stimuli that trigger craving and relapse in adolescents vs. adults. In general, adolescent rats display similar or stronger cocaine CPP and require more time to extinguish CPP behaviors relative to adults. Adolescent rats also self-administer equivalent or greater amounts of cocaine than adults and extinguish operant responding to a similar extent. With respect to relapse triggers, adolescents appear to be more sensitive to stress and contextual cues relative to discrete cues predictive of cocaine. An important consideration for future research is that adolescent rats may display comparable drug-taking behavioral profiles as adults but, consistent with work in this Research Topic, the impact of drug-induced changes on behavior or hormone function may only manifest later in life or after protracted abstinence.

In conclusion, the present Research Topic highlights sex- and/or age-specific novel neurochemical and cognitive factors that may affect susceptibility to SUDs. The studies suggest female vulnerability to stimulant use, whereas males may be more vulnerable to the later impacts of alcohol use. Adolescence is observed to be a vulnerable period for substance exposure, with immediate consequences on anxiety and cognition during adolescence as well as enduring consequences on cognition later in life. These findings emphasize the importance of sex- and age-specific research in behavioral neuroscience to promote future preclinical studies highly relevant to deepen our understanding of SUDs.

## Author contributions

CO: Writing – original draft, Writing – review & editing. JK: Writing – original draft, Writing – review & editing. AA: Writing – original draft, Writing – review & editing.
